# Automatic loop centring with a high-precision goniometer head at the SLS macromolecular crystallography beamlines

**DOI:** 10.1107/S0909049511011848

**Published:** 2011-05-14

**Authors:** Anuschka Pauluhn, Claude Pradervand, Daniel Rossetti, Marco Salathe, Clemens Schulze-Briese

**Affiliations:** a Paul Scherrer Institut, CH-5232 Villigen, Switzerland

**Keywords:** beamline automation, sample centring, goniometer head

## Abstract

Automatic loop centring has been developed as part of the automation process in crystallographic data collection at the Swiss Light Source. The procedure described here consists of an optional set-up part, in which the background images are taken, and the actual centring part. The algorithm uses boundary and centre-of-mass detection at two different microscope image magnifications. Micromounts can be handled as well. Centring of the loops can be achieved in 15–26 s, depending on their initial position, and as fast as manual centring. The alignment of the sample is carried out by means of a new flexural-hinge-based compact goniometer head. The device features an electromagnet for robotic wet mounting of samples. The circle of confusion was measured to be smaller than 1 µm (r.m.s.); its bidirectional backlash is below 2 µm.

## Abstract 2

An automated loop-centring program and a high-precision goniometer head used at the Swiss Light Source are described.

## 1. Introduction

The speed of data collection for crystallographic experiments has increased tremendously during recent years. This is due to optimized radiation sources as well as faster detectors (Broennimann *et al.*, 2006 ▶; Henrich *et al.*, 2009 ▶). Automatic sample changers are in use to further reduce the time needed for an experiment (Cipriani *et al.*, 2006 ▶; Soltis *et al.*, 2008 ▶), and an increasing number of facilities offer possibilities for remote data collection (González *et al.*, 2008 ▶; Gabadinho *et al.*, 2008 ▶; Smith *et al.*, 2010 ▶). All these improvements and advanced levels of automation leave in many cases the precise positioning of the crystal sample as the slowest step of the entire process. Moreover, the large increase in sample production resulting from high-throughput crystallization facilities requires fast screening methods. Thus, several programs for automatic centring have been developed in order to aid users in faster data collection (Lavault *et al.*, 2006 ▶; Andrey *et al.*, 2004 ▶; Pothineni *et al.*, 2006 ▶; Jain & Stojanoff, 2007 ▶).

In many cases unfavourable and varying light conditions as well as iced and otherwise opaque samples make the detection of the crystal itself quite challenging. Illumination with ultraviolet (UV) light has been proposed to support crystal detection (Pohl *et al.*, 2004 ▶; Vernede *et al.*, 2006 ▶) by taking advantage of the fluorescence emission of most proteins (those containing aromatic ring structures, like tryptophan, tyrosine and phenylalanine) when exposed to light of wavelengths between 250 nm and 290 nm. This, however, means significant additional effort in the experimental set-up and requires precise control of the exposure time in order to avoid damage. UV-induced structural changes have been described and used for phasing, for example by Nanao & Ravelli (2006 ▶) ▶.

At the Swiss Light Source (SLS), currently three beamlines are in operation for macromolecular crystallography. X06SA and X10SA are undulator beamlines, and X06DA uses a superbending magnet as photon source. At X06SA and X10SA, PILATUS 6M (http://www.dectris.com/) pixel detectors are used for data collection, featuring frame rates of up to 12.5 Hz. X06DA and the microfocus diffractometer at X06SA both employ MAR225 CCDs (http://www.rayonix.com/). In this paper we describe a simple procedure that is used at the SLS beamlines to reduce the time spent for an experiment by assisting in centring of the micromount or loop in which the crystal sample has been mounted. Only two angles (0° and 90°) are used in a first step at low magnification, and image processing is kept as simple as possible.

The requirements for the sample stages with respect to precision are high: for small beams and sample sizes of the order of tens of micrometres in particular, the sphere of confusion of a positioning device must not exceed a few micrometres. We also introduce a new goniometer head which meets these demands.

## 2. The centring procedure

The centring algorithm is based on centre-of-mass determination, and often the crystal itself, not only the loop, is centred after the procedure. By employing different microscope zooms the procedure makes optimal use of the particular settings at the beamline. In brief, the centring program consists of the following steps, which will be described in more detail below:

(i) Set goniometer angle to 0°: (*a*) retrieve the JPEG images from the server; (*b*) de-noise and convert to black and white; (*c*) find the centre of mass; (*d*) move the motors in order to place the sample at the beam position.

(ii) Set goniometer angle to 90°.

(iii) Repeat the above steps.

(iv) Change the magnification and repeat.

Loop movement is deliberately stopped and manual intervention is requested if the calculated movement would exceed a distance larger than 2.3 mm. This is a rather conservative limit that sometimes stops the procedure prematurely but is intended to prevent samples from moving out of the cryostream.

In order to have a large field of view for sample detection, the entire procedure is first executed at low magnification. Afterwards it is repeated at high magnification and the centre of mass of the loop placed in the X-ray beam.

**Figure 1:**
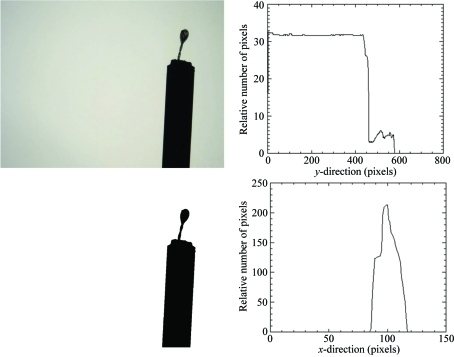
First image-processing steps: the image is converted to black and white (left), and the boundaries are found using projections of the image onto the horizontal and vertical directions (right).

The images are retrieved *via* an http server, communication with the motors is realised *via* an EPICS (Experimental Physics and Industrial Control System, http://www.aps.anl.gov/epics/) interface, and the algorithm has been implemented using IDL (Interactive Data Language, http://www.ittvis.com/). The first image-processing steps (depicted in Fig. 1 ▶) are the conversion to black and white and determination of the region of interest. The discrimination between loop and background is performed *via* a threshold, determined by comparison of the histogram of the background only (*i.e.* an image without the loop) and the histogram of the image containing a sample. Similarly, a threshold is used to separate pin and loop in the projections of the black and white images (see right-hand side of Fig. 1 ▶). From the vertical and horizontal projections the region of interest, *i.e.* the loop with the pin removed, is determined.

**Figure 2:**
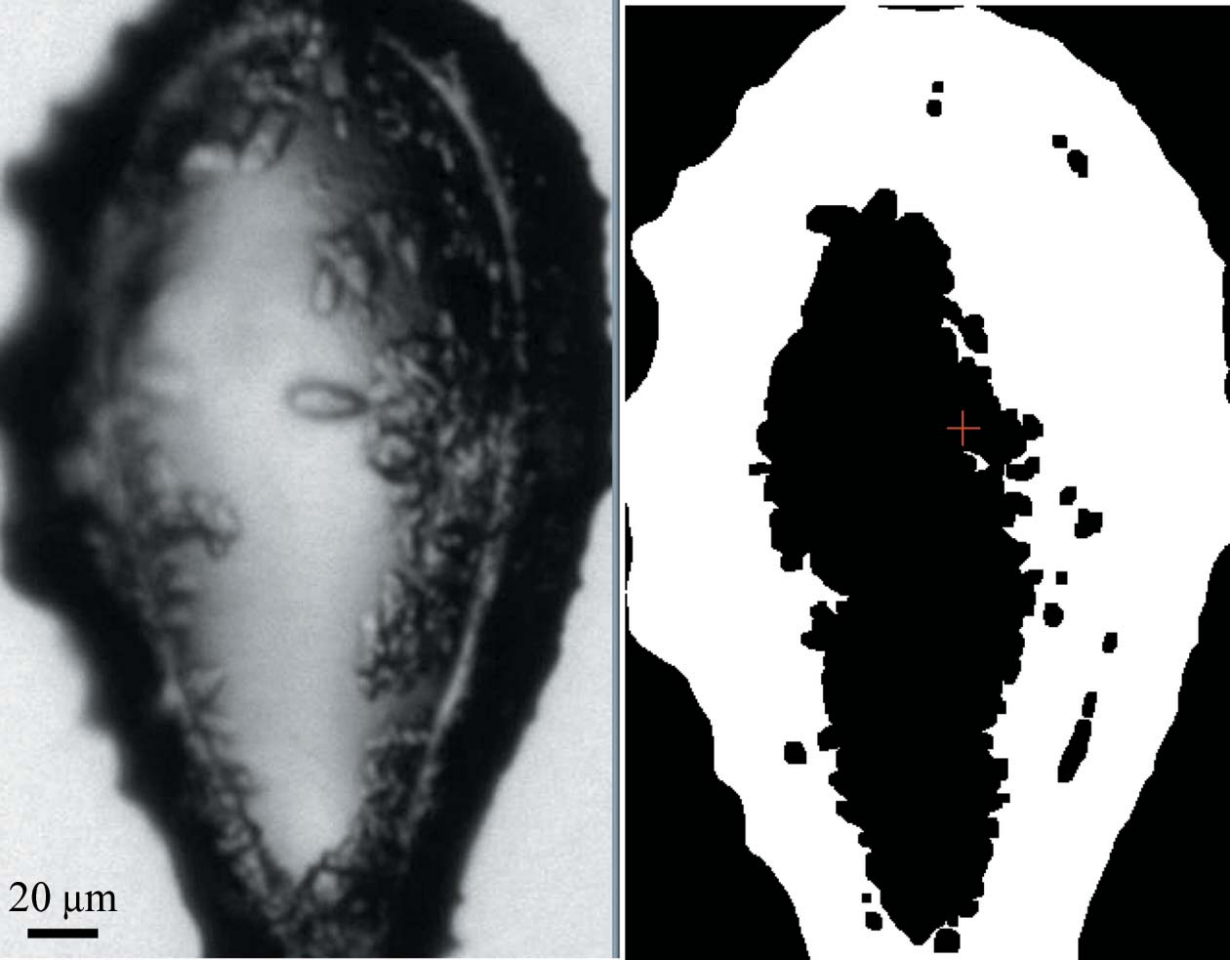
Image processing: determination of the centre of mass; shown are images at high magnification in greyscale (left) and after conversion to black and white (right).

Fig. 2 ▶ shows the region of interest determined in the first step, and the centre of mass of this part of the image which is then assumed to contain mostly loop and crystal.

**Figure 3:**
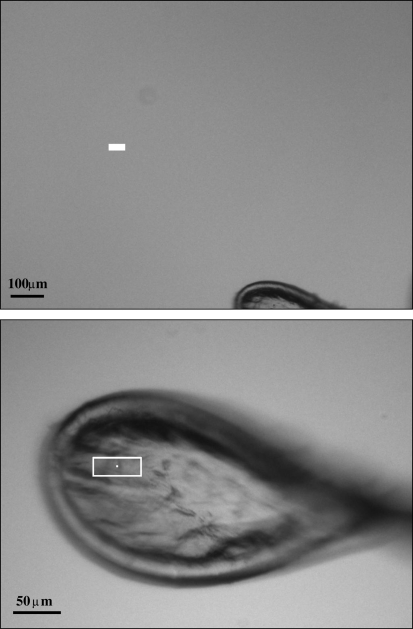
Initial (top) and final (bottom) positions (the latter after 15 s) for a test loop at beamline X10SA. The rectangles mark the beam position.

The procedure is robust and successfully centres loops which are located partly outside the field of view. An example is shown in Fig. 3 ▶.

At the beginning of the centring program the image is checked for information content (the entropy of a part of the image is calculated), and if no loop is detected (*e.g.* the sample is outside the field of view) the user is requested to re-adjust the loop and the program is interrupted.

## 3. Experimental set-up

### 3.1. The Flexor positioning system

**Figure 4:**
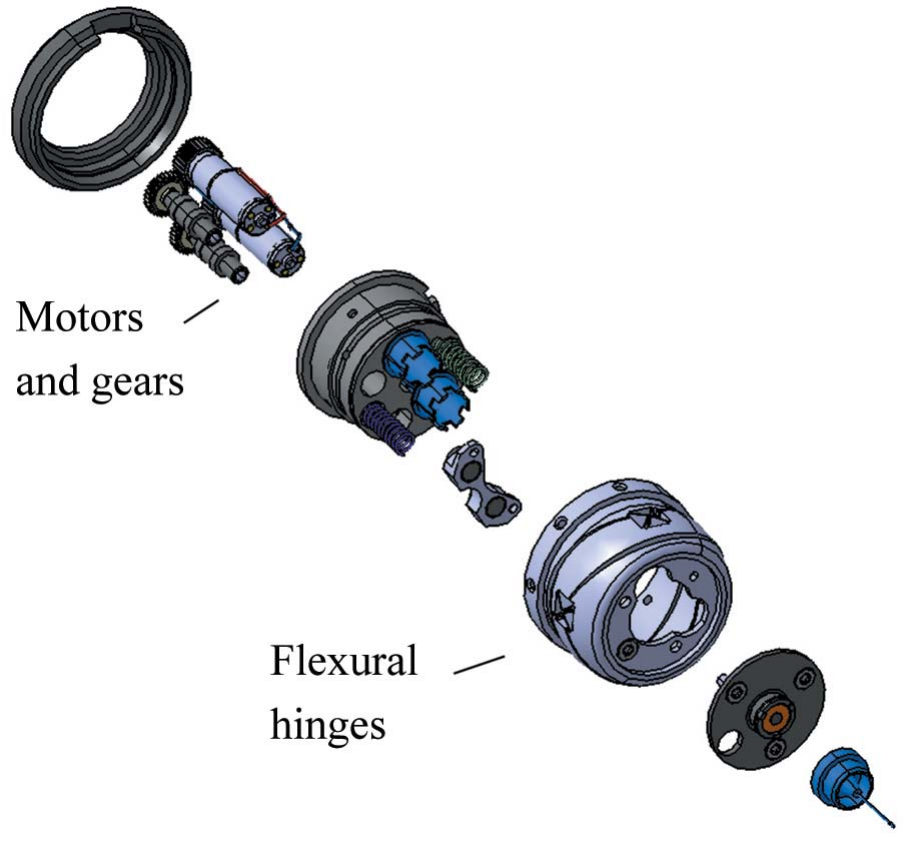
Construction view of the schematics of the Flexor positioning head.

**Figure 5:**
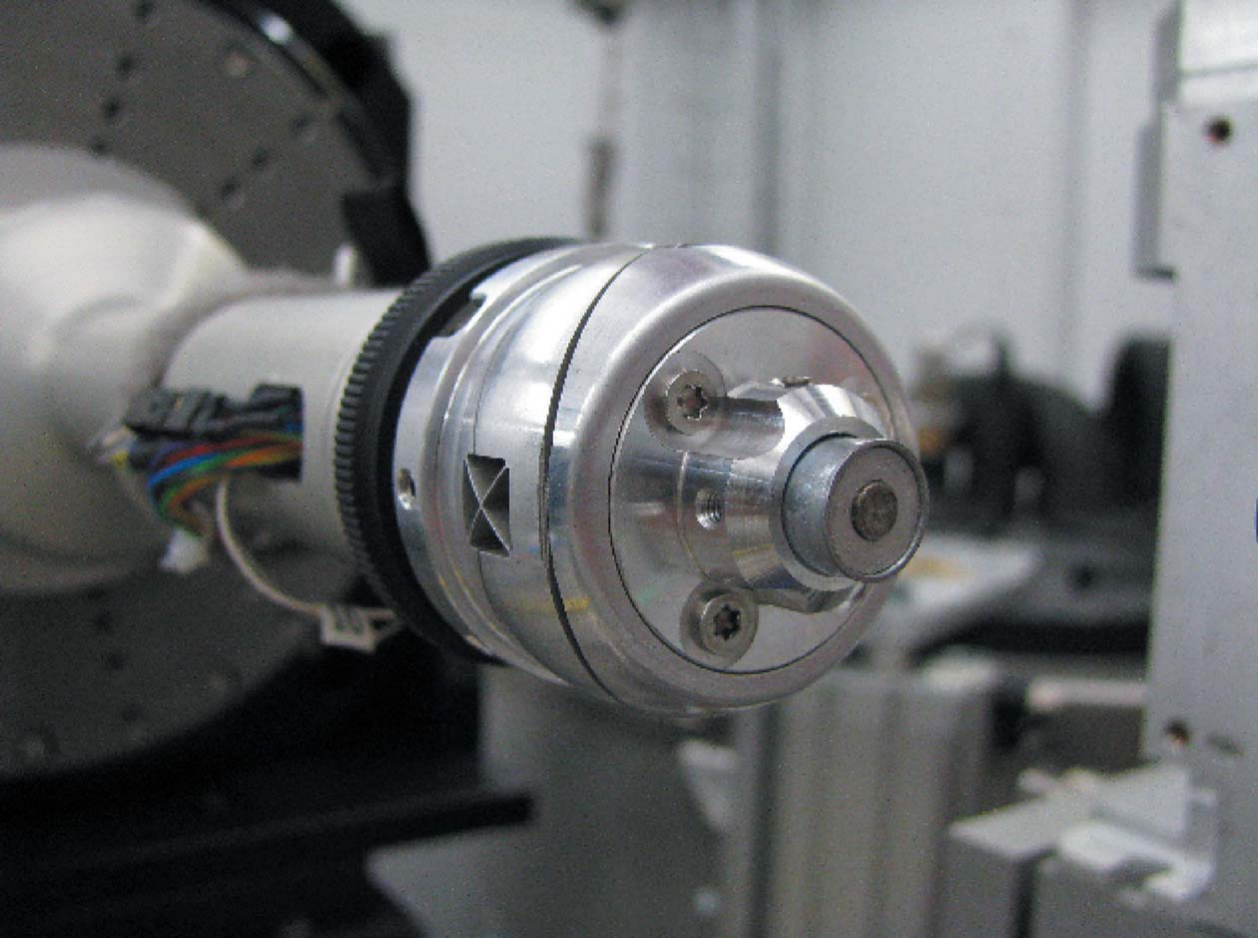
The Flexor head in the experiment environment at the beamline X06DA.

The sample stage employed at the SLS beamlines is a *y*,*z*-positioning device based on flexural hinges (the so-called ‘Flexor head’). Flexural bearings offer several advantages: they are free from friction and backlash, and they do not require lubrication. Moreover, they can be manufactured in a very compact manner (Henein, 2000 ▶). The compact and precise sample-positioning device which can be combined with an electromagnet was originally developed for use with a Kappa goniometer (Ulmer, 2007 ▶; Salathe, 2010 ▶). Currently it is mounted on the *x*-translation on top of a single ϕ-rotation table of an air bearing (ABR1000, http://www.aerotech.co.uk/). Fig. 4 ▶ shows the construction outline of the positioning head, and Fig. 5 ▶ presents a view of the device within the diffractometer assembly at beamline X06DA.

### 3.2. Principle

The Flexor construction positions the sample by tilting the pin base in two perpendicular directions. The assembly is held tense and stabilized using springs. The drive shafts are driven by step motors. The resulting motion in the *y* and *z* directions is dependent on the length of the pin and is calculated automatically from the absolute position of the *x*-translation.

The main specifications of the device are: maximum tilt angle: ±3.5°; maximum range: ±2 mm (with 18 mm pin); resolution: 0.3 µm; speed: 0.3 mm s^−1^. The diameter of the device is 38 mm; its overall length (including the magnet) amounts to 50 mm.

### 3.3. Precision

In all measurements shown the position of the needle (pin of size 1 µm) was automatically determined using the high-resolution images in the corresponding set-ups of microscope and cameras at the beamline or in the laboratory.

The Flexor exhibits high positional stability. In the laboratory test-bench measurements all tested Flexor devices showed a peak-to-peak deviation of around 2 µm with standard deviation σ = 0.5 µm (Salathe, 2010 ▶). In the real experimental beamline environment the additional effects of the arm of the sample stage and the fixations add to the imperfections, and the resulting peak-to-peak deviation amounts to 3 µm, with σ ≤ 1 µm.

**Figure 6:**
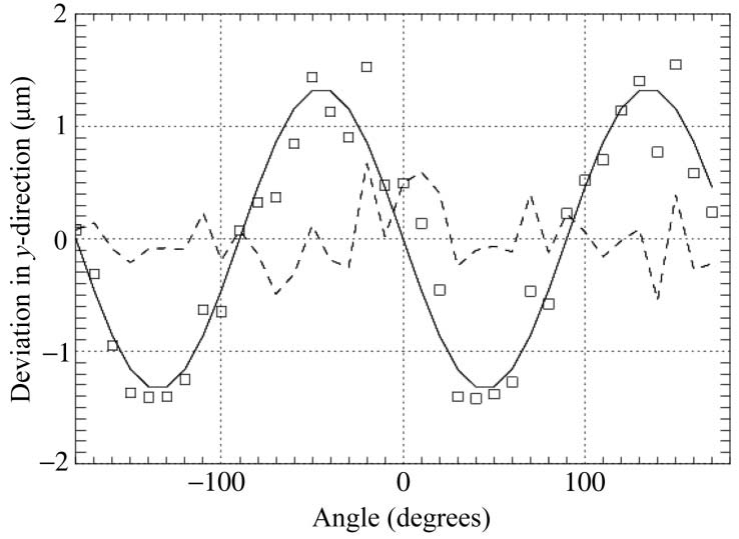
The peak-to-peak variation along the vertical direction of the Flexor head positioning, given by the position of the 1 µm needle during a 360° rotation. Shown are the measurements of the needle position (squares), together with a sine-fit (solid line) and the residual (dashed line), at beamline X06DA.

**Figure 7:**
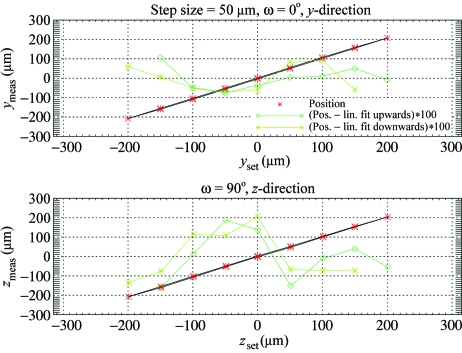
The bidirectional accuracy of the Flexor head motion. Shown is the measured position of a needle (1 µm size) *versus* the nominal (red stars), together with the differences to the linear fits (green stars and squares). The latter values have been enlarged by a factor of 100.

Fig. 6 ▶ shows the position of a needle during a 360° rotation. The double-sine-curve characteristic of the Flexor is due to a slight internal asymmetry, as, owing to space limitations, only a single spring is used to provide the restoring force. After subtraction of the sine-fit, the residual σ is less than 0.3 µm, and thus in the range of the still measurements (Salathe, 2010 ▶). The reproducibility of the Flexor positioning has been tested *via* a ‘hysteresis curve’ measurement. Starting from a centre position one of the motors (*y*- or *z*-direction) is moved in 50 µm steps upwards to +200 µm, then downwards to −200 µm and back to the centre again. The other direction is kept fixed, and the measurement is repeated *vice versa*. Fig. 7 ▶ displays the position of a Flexor-mounted needle during this cycling of the *y*- and *z*-motors. Also plotted are the differences to the linear fits to the upward and downward movements with a 100-times enlarged scale, indicating that the positioning is accurate to 2 µm.

### 3.4. Loop centring at the beamline

All three beamlines have different sample environments which vary in terms of illumination, field of view and microscopes; therefore the program has been adapted accordingly. We have set up a standard testing procedure, with different initial positions.

**Figure 8:**
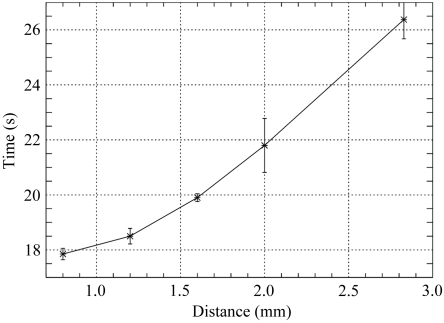
The time to bring the loop into the centre of rotation (beam position) at beamline X06SA *versus* distance (in the plane of view, *i.e.* the *x*–*z* plane only). The values are averages over four inital positions.

The time required for centring is dependent on the initial distance of the sample to the centre of rotation/beam (apart from the mechanical set-up, like the speed of the motors), *cf.* Fig. 8 ▶.

#### 3.4.1. X06SA

The viewing system at beamline X06SA comprises a microscope (Leica Z16) with motorized zoom and CCD (GRAS-14S5C, Point Grey Research) installed below the sample. The field of view of the microscope at lowest magnification is 3 mm, and at highest it is 500 µm. The sample is back-illuminated by a condenser lamp mounted above it. The size of the focused X-ray beam is 85 µm by 10 µm. Loop centring takes 15–27 s.

#### 3.4.2. X06DA

The viewing system at beamline X06DA is similar to that of X06SA with respect to the optics (Leica Z16 microscope and CCD FLEA-HIBW from Point Grey Research). However, the sample image is deflected 90° downwards by a prism system with a collimator where the X-ray beam passes through in order to obtain an on-axis view. The illumination is provided by two LEDs. Owing to the rather large focal depth of the microscope, at low magnification a large part of the image is occupied by the blurred image of the collimator. The latter can be removed by subtracting a background file. This, however, might affect part of the loop structure when it is situated right in front of the collimator. Additionally, subtracting a background image introduces speckle noise, mainly due to slightly different light conditions at different times as a result of the optical distortions produced by the cryostream. Several techniques have been tested in order to reduce the noise in the images, such as morphological closing or simple histogram byte-scaling (*cf.* for example Gonzales & Woods, 2008 ▶). The latter has turned out to be fast and reliable. The beam size at X06DA is 80 µm by 45 µm. Loop centring takes 21–26 s.

#### 3.4.3. X10SA

At beamline X10SA the sample is also viewed on-axis as the beam passes through the centre of the microscope objective lens (Navitar Zoom-6000), with a CCD similar to that used at X06DA. The sample is back-illuminated by a Koehler-type light source. Here, the collimator is motorized and can be retracted during sample centring. However, this set-up has the disadvantage of a largely reduced field of view (1200 µm lateral extension at lowest and 300 µm at maximum magnification). This makes the initial sample location a challenge for unusual loop lengths and bent shapes. In order to increase the field of view to deal with these problems, a second camera with fixed zoom has been installed above the set-up (‘top cam’, with a lateral field of view of about 11 mm). The size of the X-ray beam is 50 µm by 10 µm. Loop centring takes 15–30 s.

Fig. 8 ▶ shows the time required to centre the sample *versus* the distance to the beam coordinates for beamline X06SA. It should be noted that the adjustment of the microscope magnification (zoom motor) takes 7 s. The motor movements contribute roughly 1 s per 0.25 mm. The rotation is performed at a speed of 180° s^−1^. Initially the cryojet is moved by 5 mm out of the image, which also takes roughly 5 s. Furthermore, additional centring steps at 45° and 135° have been included. This shows that there is basically no overhead produced by the program.

#### 3.4.4. Loop centring and sample changer operation

**Figure 9:**
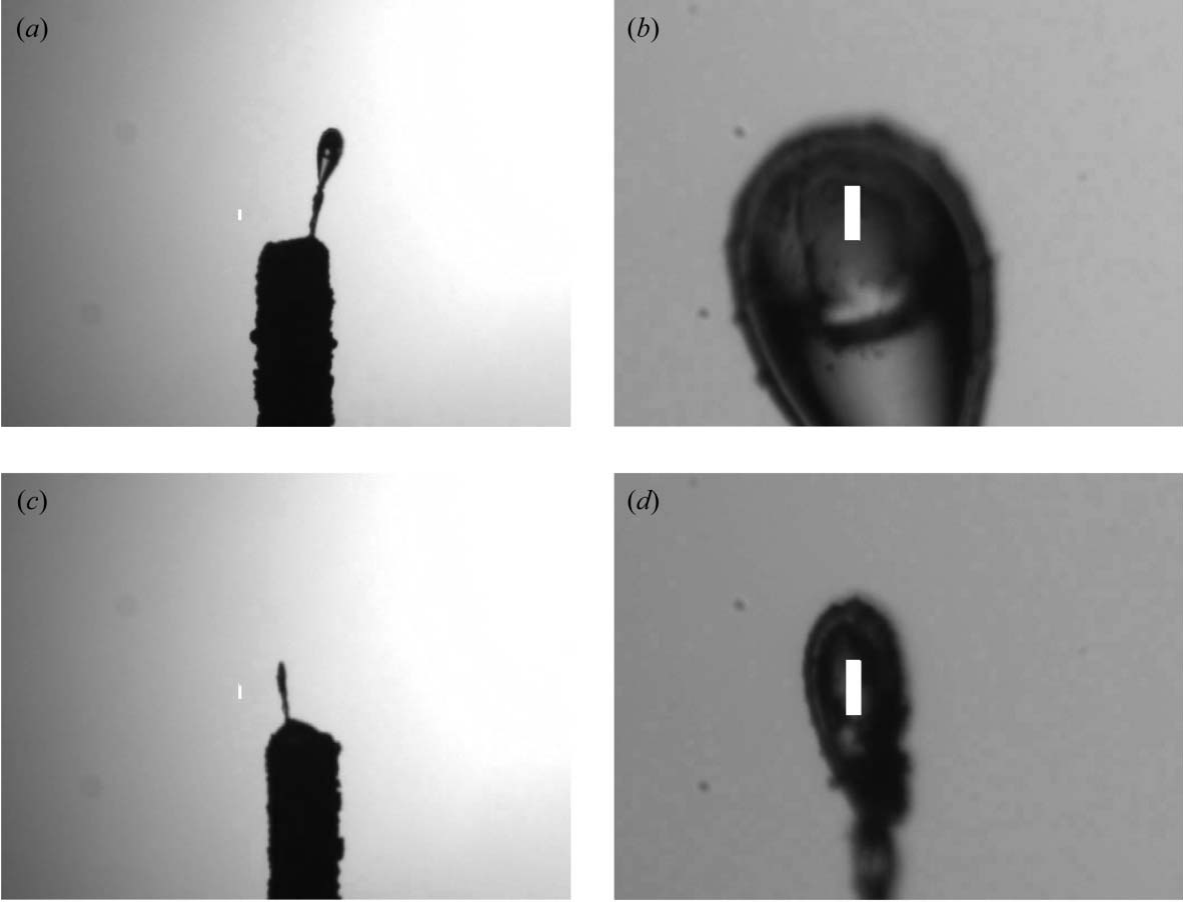
Initial (*a* and *c*) and final (*b* and *d*) positions of samples during a long-term test with the CATS sample changer at X06SA. The white rectangles mark the beam position and size (85 µm by 10 µm). In (*a*) and (*c*) an inhomogeneous illumination is visible; nevertheless the loop could be centred.

All three SLS beamlines have been equipped with automatic sample changers of CATS type (Jacquamet *et al.*, 2009 ▶). During long-term tests of the sample changer at beamline X06SA, the loop-centring program was used to position the sample in the beam after mounting. Snapshots of the sample before and after centring were made automatically. Fig. 9 ▶ shows two of the results of loop-centring during CATS operation at X06SA; centring times varied between 19 s and 26 s.

#### 3.4.5. Limitations

The software is sensitive to changes in the illumination (lamp brightness, angle) as well as a possible offset in the microscope axis from the vertical (or, more generally, to misalignments of the microscope axis). The first produces shadows and ‘false positives’ shifting the centre of mass artificially away from the crystal. In Fig. 9 ▶ the images show a misalignment of the condensor of the illumination, generating a gradient in brightness across the image. The latter results in an offset for the centres of rotation at different magnifications. If it is kept stable, this can be taken into account and corrected for. An accurate alignment of the microscope and camera system as well as a homogeneous illumination of the sample field of view by the lamp condensor are thus a prerequisite for automatic loop centring.

## 4. Summary

The described loop-centring procedure is stable and fast, in the sense that it does not lead to temporal overheads compared with manual centring. Depending on the initial position, loop centring can be achieved in 15–26 s. Centring within 20–30 s can be achieved even if additional angles (*e.g.* 45° and 135°) are selected for a final centring at maximum magnification, enhancing the probability of not only moving the loop but also the crystal into the centre of rotation. Success rates are around 98% at beamline X06SA. This was deduced as the ratio of tests with successfully centred loops and micromounts (242) to the total number of tests (247) at X06SA with initial conditions in various positions. Snapshots were taken automatically at initial and final positions. Icy samples do not pose a problem for the loop centring; they do, however, reduce the chance of centring on the crystal itself. Causes for failure are mainly deficient illumination or samples being too far off the centre that the program does not start. The program has been applied in combination with an automatic sample changer. It is foreseen to promote further automation at the beamline using automatic loop centring in combination with diffraction-based alignment (see, for example, Song *et al.*, 2007 ▶). The goniometer head used at the beamlines features a small circle of confusion of less than 1 µm (r.m.s.), compatible with beam dimensions. A further improvement will be implemented by using piezo-based translation stages, in order to cope with smaller beam sizes created by use of apertures.
